# Supplementary figures and images for: Aldh inhibitor restores auditory function in a mouse model of human deafness
Source: PLoS Genet. 2020 Sep 24;16(9):e1009040. doi: 10.1371/journal.pgen.1009040 (PMC7553308; doi:10.1371/journal.pgen.1009040)

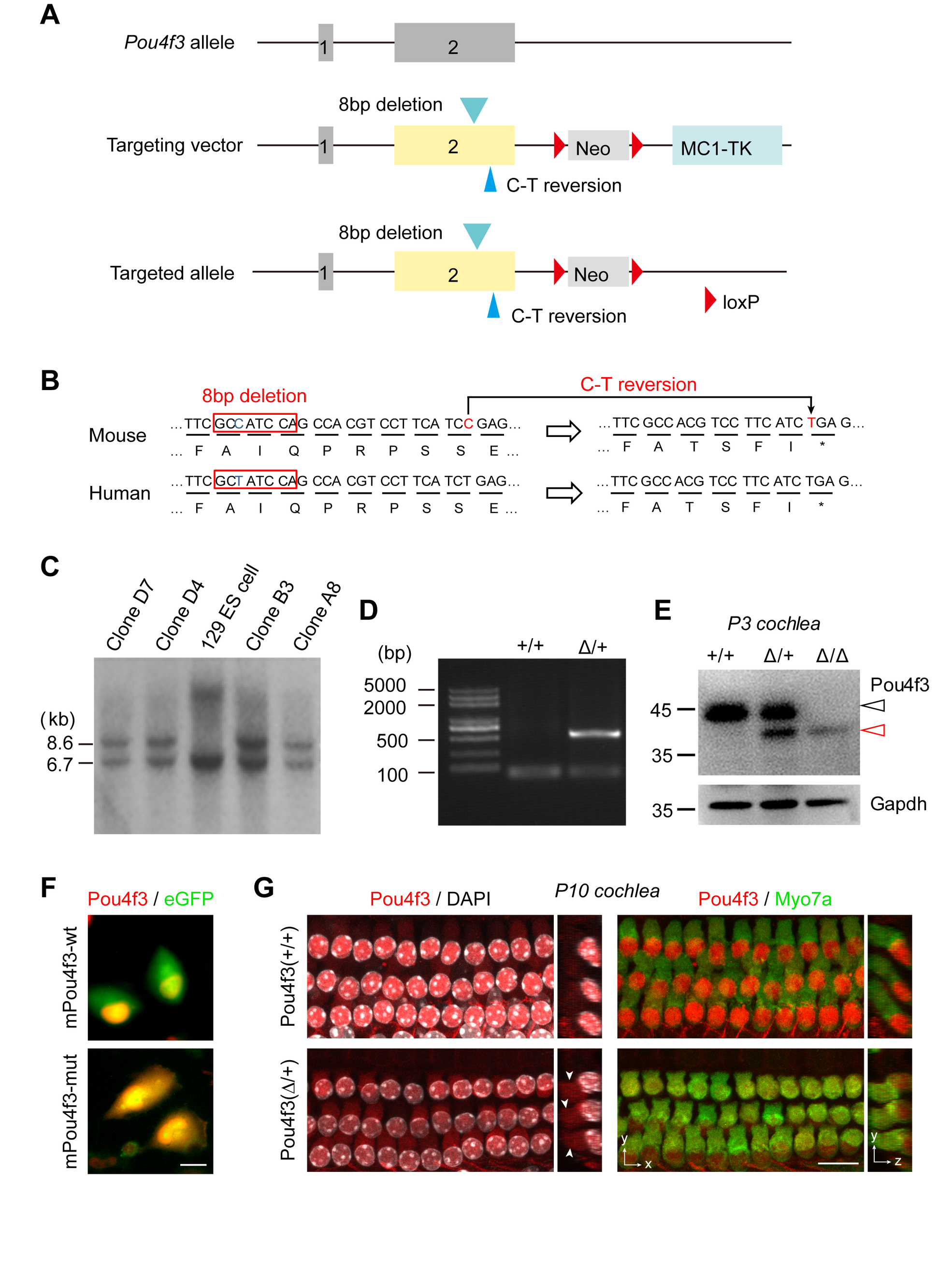

Supplement: S1 Fig — (A) Schematic representation of the Pou4f3 knockin strategy using Pou4f3 gene with the 8 bp deletion and C-T reversion. (B) Nucleotide and amino acid sequences of wildtype and mutant Pou4f3. Red boxes indicate the 8bp deletion identified in DFNA15 patients. C-T reversion was applied to generate the premature stop codon in the mouse version of mutant Pou4f3. (C) DNA isolated from chimeric and wild-type mice tails was digested with EcoRV and analyzed by Southern blot. The wild-type and mutant alleles yield 6.7 kilobase (kb) and 8.6kb fragments, respectively. (D) PCR genotyping for the Pou4f3(Δ/+) mice. A band of 542bp size can be detected in the genomic DNA from the Pou4f3(Δ/+) mice. (E) Western blot analysis for Pou4f3 protein from cochlear sensory epithelia of P3 mice. The black open arrow indicates the wildtype Pou4f3 in both control and Pou4f3(Δ/+) mice while red open arrow shows the truncated Pou4f3 protein. (F) Localization of the wildtype and mutant mouse Pou4f3 in HeLa cells. Wildtype of mutant Pou4f3 were cloned to pIRES-eGFP plasmid and expressed in HeLa cells. eGFP was localized to both cytoplasm and nucleus. Scale bar was 10 μm. (G) Expression and localization of Pou4f3 protein in outer hair cells of P10 cochleae from Pou4f3(+/+) and Pou4f3(Δ/+) mice. Arrow heads indicate cytoplasmic localization of the mutant Pou4f3. Scale bar was 20 μm. (TIF) [file pgen.1009040.s001.tif]

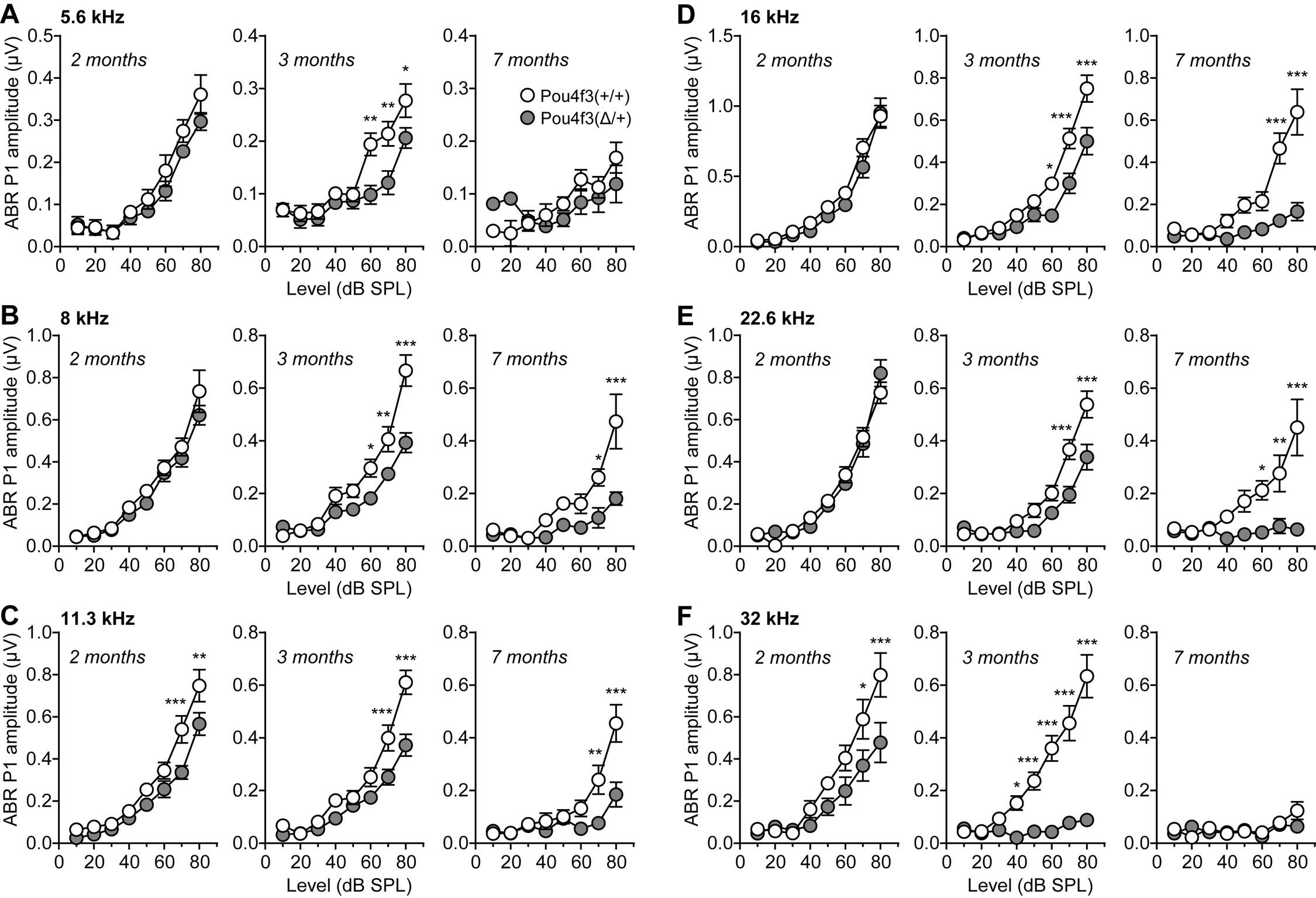

Supplement: S2 Fig — (A-F) ABR P1 amplitude growth curves of Pou4f3(+/+) and Pou4f3(Δ/+) mice at 2, 3 and 7 months of age. A, 5.6 kHz; B, 8 kHz; C, 11.3 kHz; D, 16 kHz; E, 22.6 kHz; F, 32 kHz. * P < 0.05, ** P < 0.01 and *** P < 0.001 by two-way ANOVA. (TIF) [file pgen.1009040.s002.tif]

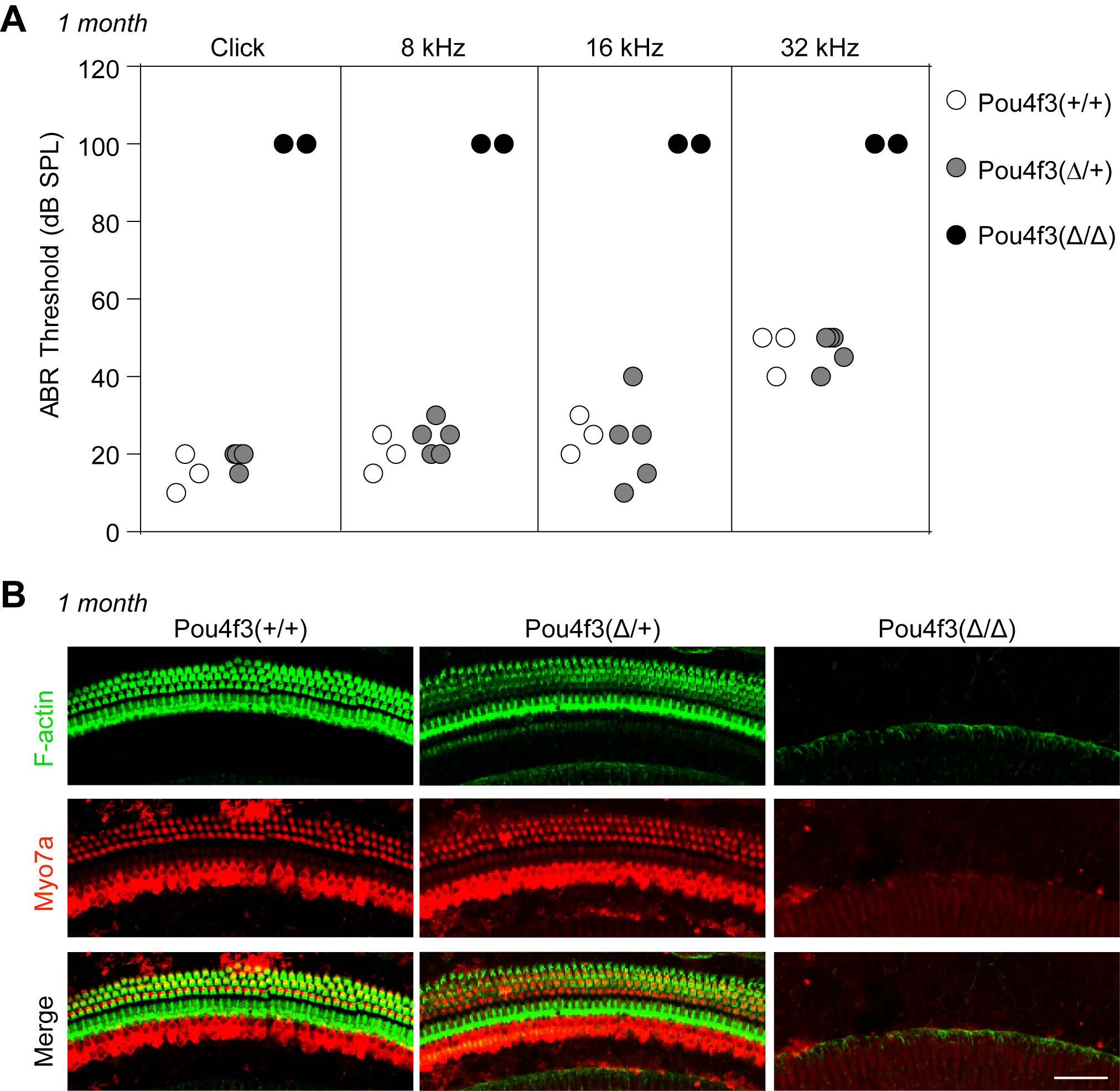

Supplement: S3 Fig — (A) ABR thresholds for click and pure tones (8, 16 and 32 kHz) of 1-month old Pou4f3(+/+) (n = 3), Pou4f3(Δ/+) (n = 5) and Pou4f3(Δ/Δ) (n = 2) mice. The two Pou4f3(Δ/Δ) mice were completely deaf without evocable ABR responses. (B) F-actin labelling and Myo7a immunostaining of the apical cochlear turn from 1 month old Pou4f3(+/+), Pou4f3(Δ/+) and Pou4f3(Δ/Δ) mice. Both OHCs and IHCs were completely lost in the sensory epithelia of Pou4f3(Δ/Δ) mice. Similar result was observed at the basal turn. Scale bars: 50 μm. (TIF) [file pgen.1009040.s003.tif]

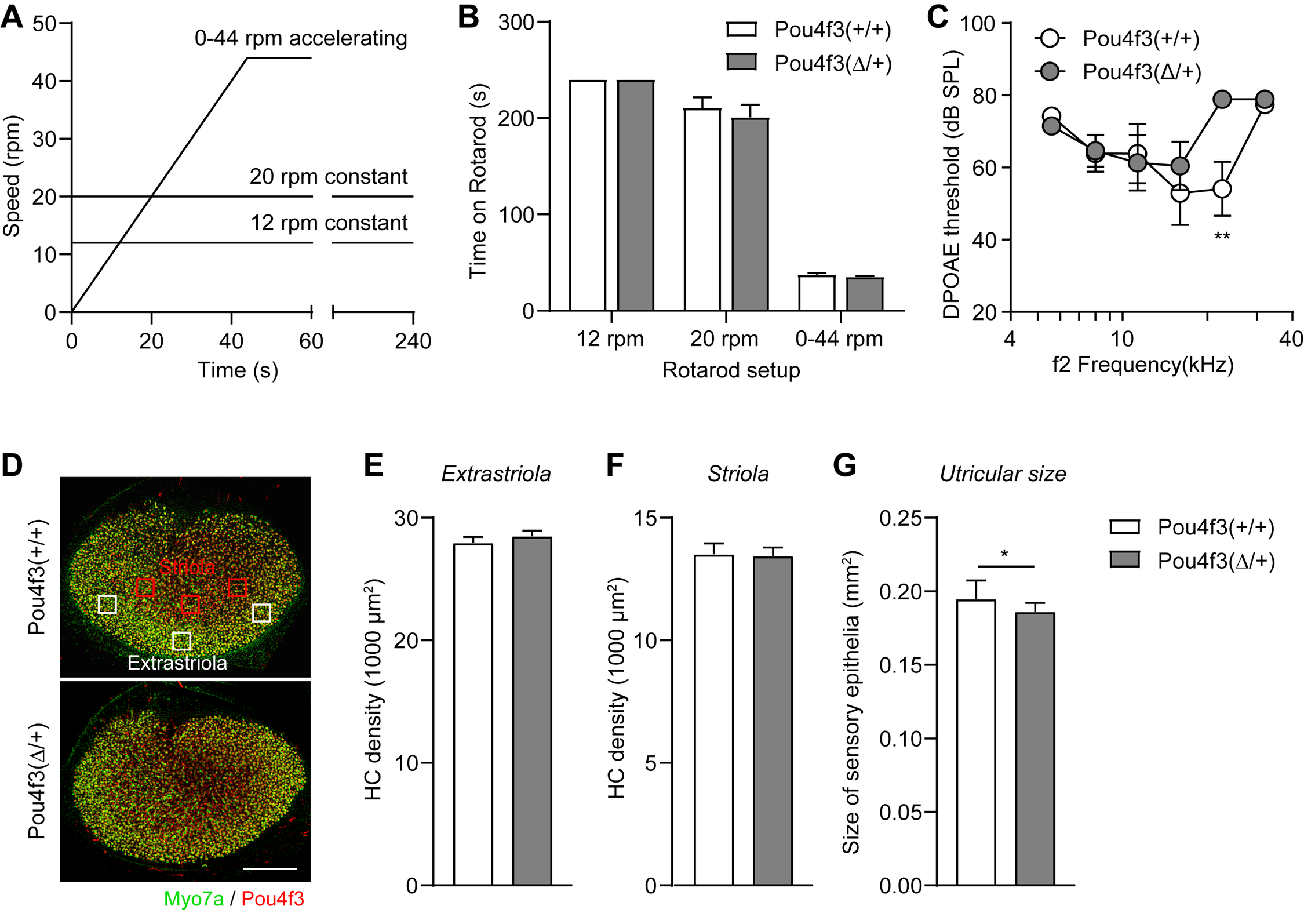

Supplement: S4 Fig — (A) Schematic representation of the rotarod testing protocols. (B) The time to fall from the rotarod of Pou4f3(+/+) and Pou4f3(Δ/+) mice. No significant difference was observed with all 3 testing protocols. n = 11–13 mice of each genotype. (C) DPOAE tests of the Pou4f3(+/+) and Pou4f3(Δ/+) mice. ** P < 0.01 by two-way ANOVA, n = 10 mice of each genotype. (D) Myo7a and Pou4f3 immunofluorescence images showing the entire utricular sensory epithelium from Pou4f3(+/+) or Pou4f3(Δ/+) mice. Squares represent high magnification samplings of extrastriolar and striolar areas. Scale bar was 100 μm. (E-F) Density of utricular hair cells in extrastriolar area (E) and striolar area (F) in Pou4f3(+/+) or Pou4f3(Δ/+) mice. (G) The surface areas of utricular sensory epithelia in Pou4f3(+/+) or Pou4f3(Δ/+) mice. * P < 0.05 by unpaired student’s t-test, n = 12–13 utricles of each genotype. (TIF) [file pgen.1009040.s004.tif]

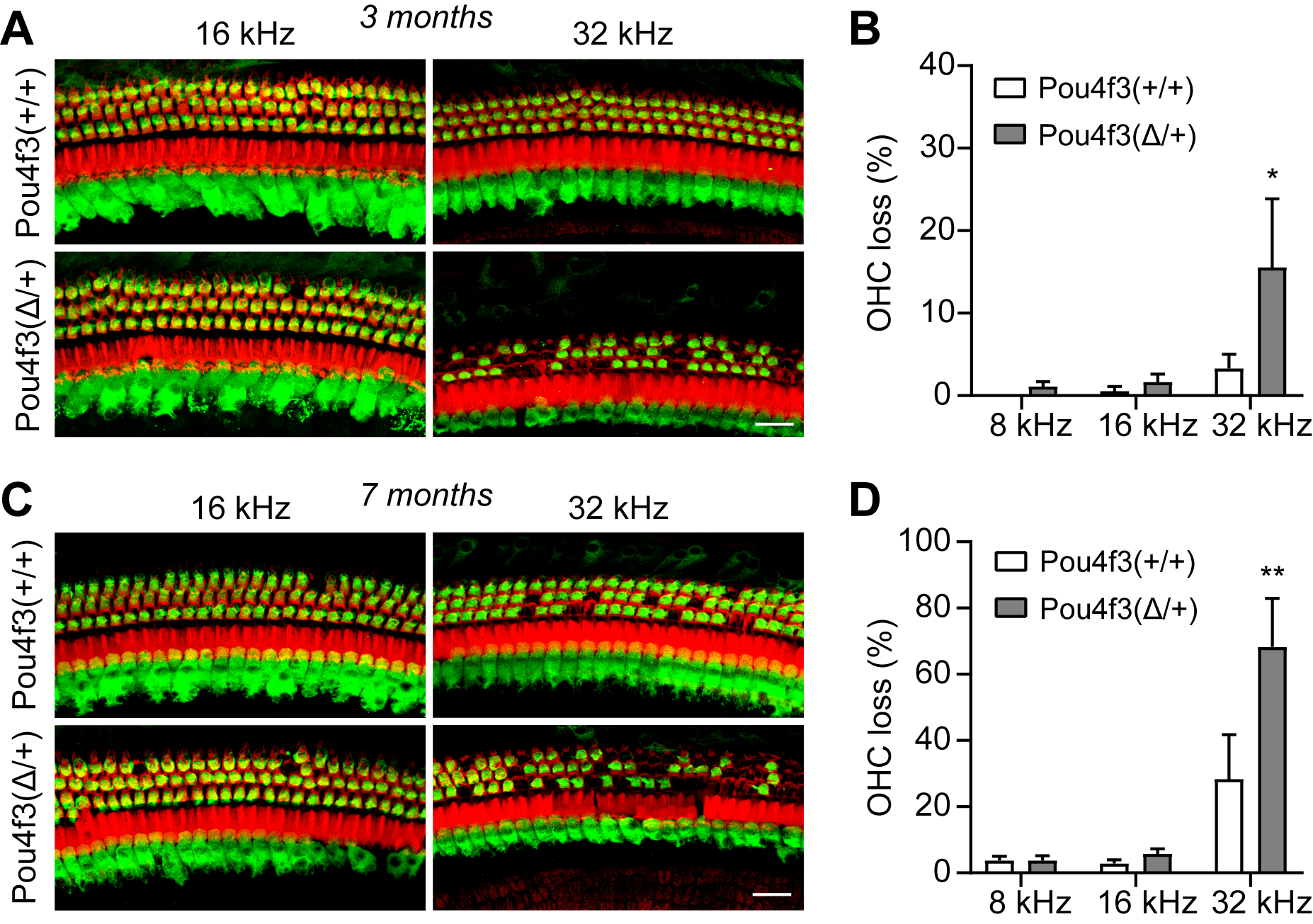

Supplement: S5 Fig — (A, C) Myo7a immunostaining images of the cochlear sensory epithelia from (A) 3 months and (C) 7 months old Pou4f3(+/+) and Pou4f3(Δ/+) mice. Hair cells and F-actin was labelled with Myo7a (green) and Rhodamine-phalloidin (red), respectively. Scale bar was 20 μm. (B, D) Percentage of outer hair cell loss in (B) 3 months and (D) 7 months old Pou4f3(+/+) and Pou4f3(Δ/+) mice. * P < 0.05 and ** P < 0.01 by two-way ANOVA, n = 3–4 cochleae of each genotype. (TIF) [file pgen.1009040.s005.tif]

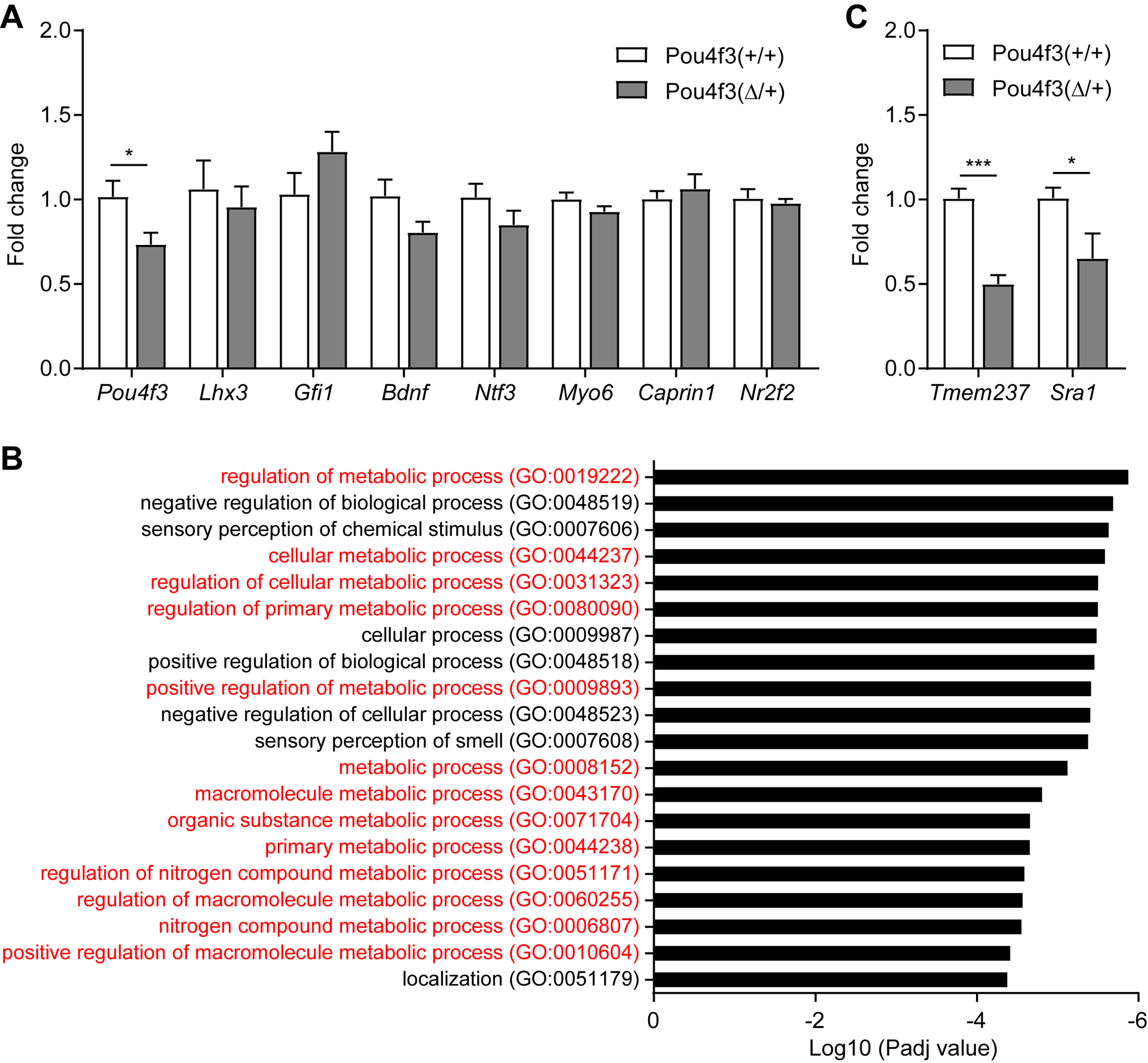

Supplement: S6 Fig — (A) Gene expression analyses of Pou4f3 and its known downstream target genes by RT-qPCR. (B) Top 20 gene ontology (GO) processes of differentially expressed genes in Pou4f3(Δ/+) cochleae. Metabolic processes were highlighted in red. Padj, adjusted P value. (C) RT-qPCR validations of selected genes identified from the RNA-seq experiment. * P < 0.05 and *** P < 0.001 by unpaired student’s t-test, n = 5 cochleae of each genotype. (TIF) [file pgen.1009040.s006.tif]

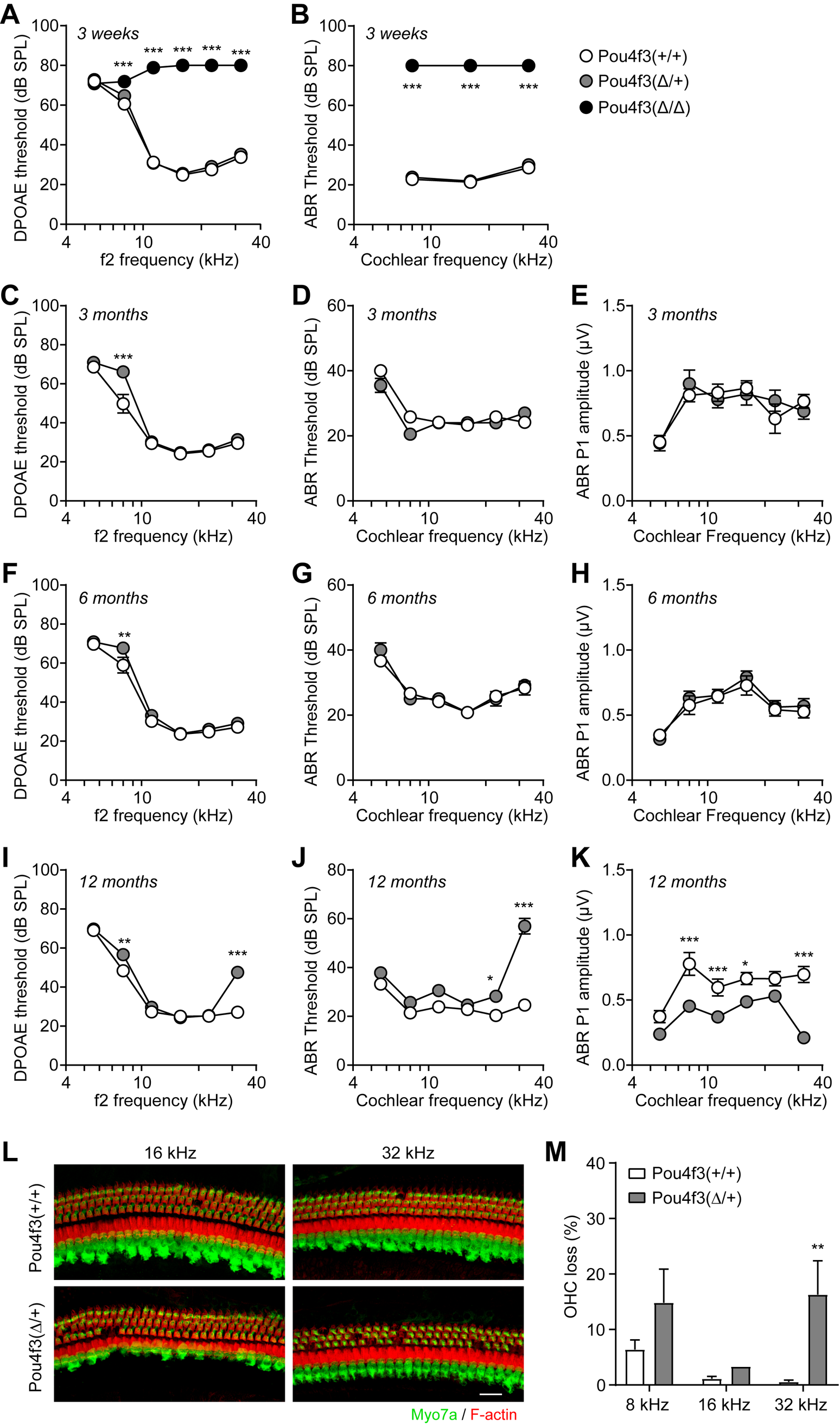

Supplement: S7 Fig — (A) DPOAE and (B) ABR thresholds of 3-week old Pou4f3(+/+) (n = 18), Pou4f3(Δ/+) (n = 21) and Pou4f3(Δ/Δ) (n = 4) mice. Pou4f3(Δ/Δ) mice were completely deaf without evocable ABR responses. *** P < 0.001 by two-way ANOVA. (C-E) 3 months (n = 6–10), (F-H) 6 months (n = 6) and (I-K) 12 months (n = 13–28) old wildtype Pou4f3(+/+) and mutant Pou4f3(Δ/+) mice were tested with DPOAE and ABR. Mice were maintained on a mixed background of C57BL/6J and FVBN. (C, F, I) DPOAE thresholds; (D, G, J) ABR thresholds; (E, H, K) ABR peak 1 (P1) amplitudes. * P < 0.05, ** P < 0.01 and *** P < 0.001 by two-way ANOVA. (L) Myo7a immunostaining images of the cochlear sensory epithelia from 12 months old wildtype and mutant mice. Hair cells and F-actin was labelled with Myo7a (green) and Rhodamine-phalloidin (red), respectively. Scale bar was 20 μm. (M) Percentage of outer hair cell loss in wildtype and mutant mice. ** P < 0.01 by two-way ANOVA, n = 3–4 cochleae of each genotype. (TIF) [file pgen.1009040.s007.tif]

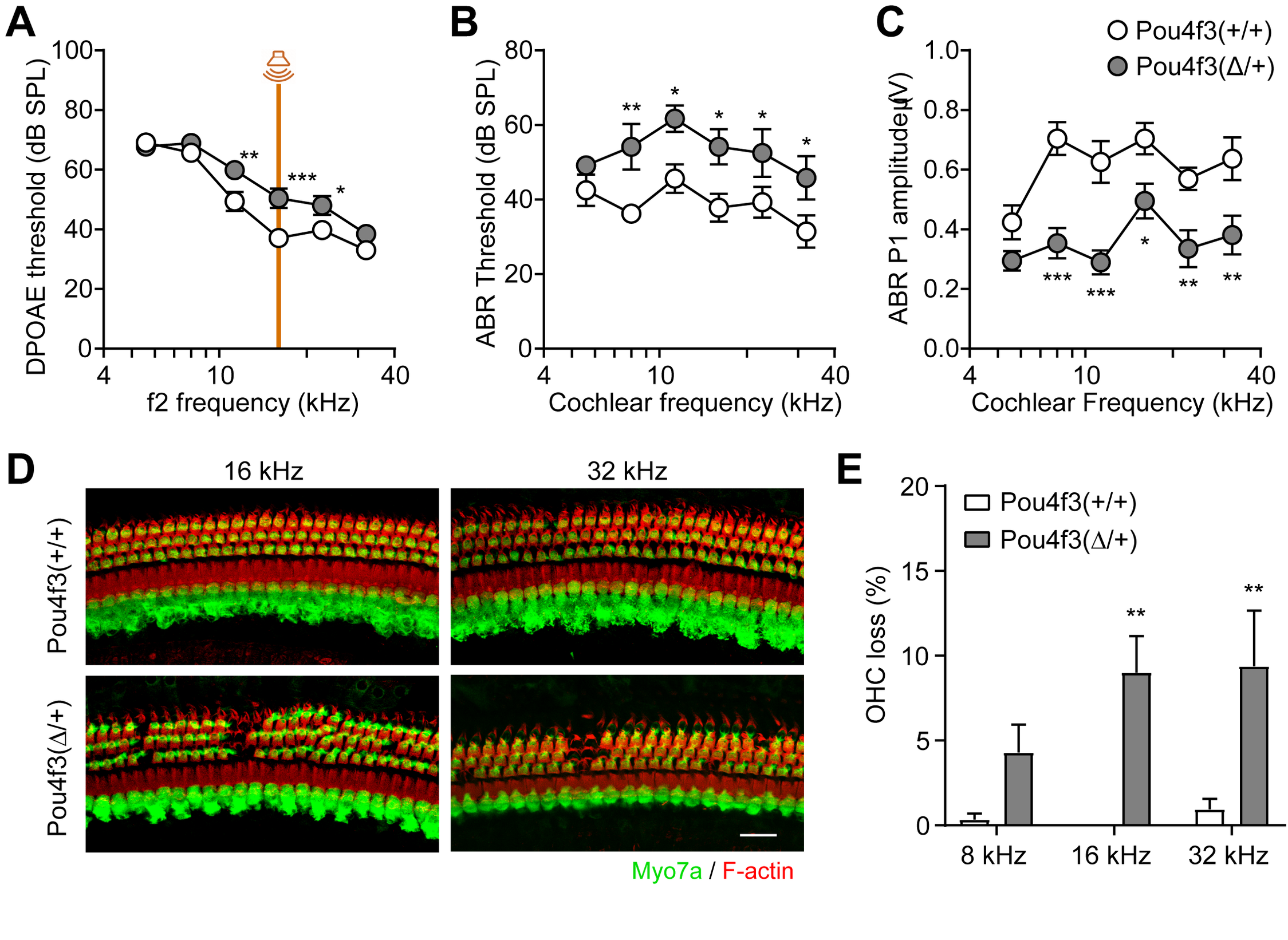

Supplement: S8 Fig — (A) DPOAE threshold (n = 12–16), (B) ABR threshold (n = 6–8) and (C) ABR P1 amplitudes (n = 6–8) of 4 months old Pou4f3(+/+) and Pou4f3(Δ/+) mice 10 days after noise exposure. Mice were maintained on a mixed background of C57BL/6J and FVBN. Symbol of speaker and vertical line indicate pure-tone noise exposure at 16 kHz, 100 dB for 2 h. * P < 0.05, ** P < 0.01 and *** P < 0.001 by two-way ANOVA. (D) Myo7a immunostaining images of the cochlear sensory epithelia from wildtype and mutant mice 10 days after noise exposure. Hair cells and F-actin was labelled with Myo7a (green) and Rhodamine-phalloidin (red), respectively. Scale bar was 20 μm. (E) Percentage of outer hair cell loss in wildtype and mutant mice 10 days after noise exposure. ** P < 0.01 by two-way ANOVA, n = 4 cochleae of each genotype. (TIF) [file pgen.1009040.s008.tif]

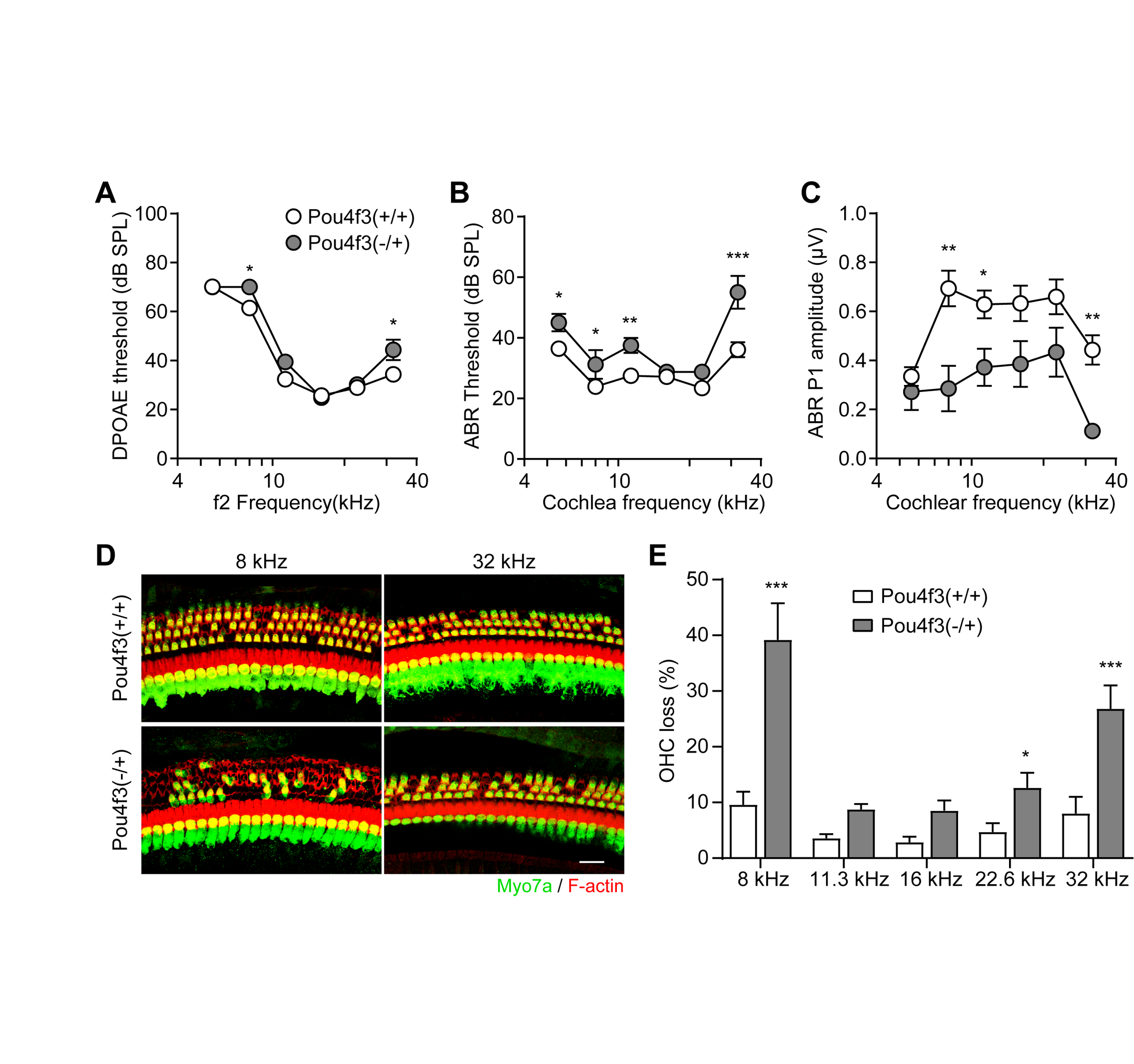

Supplement: S9 Fig — (A) DPOAE threshold, (B) ABR threshold and (C) ABR P1 amplitudes of 9 months old Pou4f3(+/+) and Pou4f3(-/+) mice. Mice were maintained on a mixed background of C57BL/6J and FVBN. * P < 0.05, ** P < 0.05 and *** P < 0.001 by two-way ANOVA, n = 4–14 mice of each genotype. (D) Myo7a immunostaining images of the cochlear sensory epithelia from 9 months old Pou4f3(+/+) and Pou4f3(-/+) mice. Hair cells and F-actin was labelled with Myo7a (green) and Rhodamine-phalloidin (red), respectively. Scale bar was 20 μm. (E) Percentage of outer hair cell loss in Pou4f3(+/+) and Pou4f3(-/+) mice at various cochlear frequencies. * P < 0.05 and *** P < 0.001 by two-way ANOVA, n = 6–10 cochleae of each genotype. (TIF) [file pgen.1009040.s009.tif]

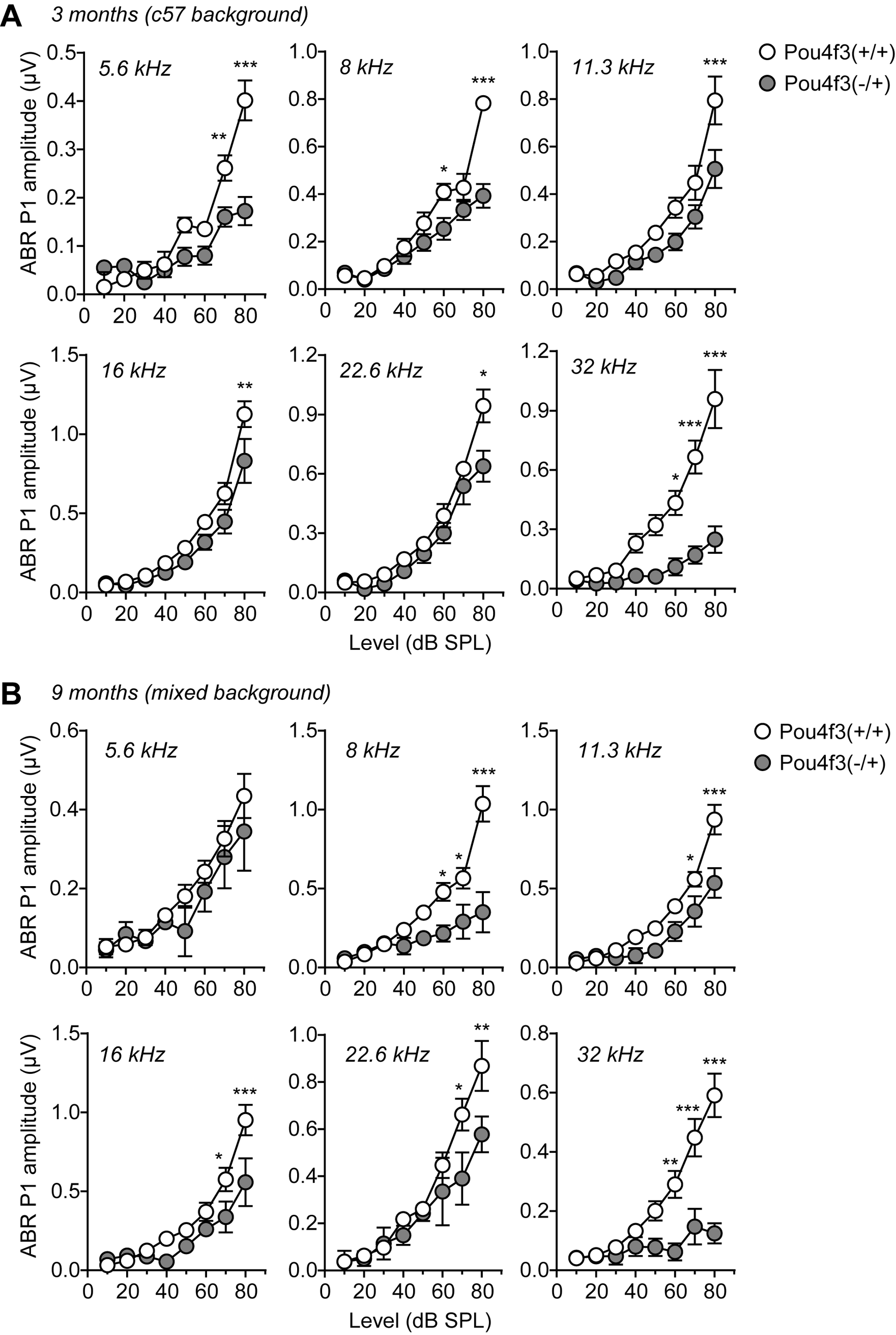

Supplement: S10 Fig — (A-B) ABR P1 amplitude growth curves of (A) 3 months old Pou4f3(+/+) and Pou4f3(-/+) mice on C57BL/6J background (n = 6–8) and (B) 9 months old Pou4f3(+/+) and Pou4f3(-/+) mice on a mixed background (n = 4–14), at 5.6, 8, 11.3, 16, 22.6 and 32 kHz. * P < 0.05, ** P < 0.01 and *** P < 0.001 by two-way ANOVA. (TIF) [file pgen.1009040.s010.tif]
