# Supplementary material for: Aldh inhibitor restores auditory function in a mouse model of human deafness
Source: PLoS Genet. 2020 Sep 24;16(9):e1009040. doi: 10.1371/journal.pgen.1009040 (PMC7553308; doi:10.1371/journal.pgen.1009040)
Supplement: S1 Table — (DOCX) [file pgen.1009040.s011.docx]

**S1 Table: Vestibular phenotypes in DFNA15 patients.**

| **Ethnicity** | **Mutation** | **Vestibular complains** | **Vestibular test** | **Reference** |
| --- | --- | --- | --- | --- |
| Israeli Jewish | c.884_891del8 | No | - | Vahara et al. 1998 |
| Dutch | c.865C.T | Yes | Reduced vestibular function in some of the family members | Collin et al. 2008 Pauw et al. 2008 |
| Dutch | c.668T.C | Yes | No substantial vestibular dysfunction | de Heer AM et al. 2009 |
| Korean | c.662_675del14 | - | - | Lee et al. 2010 |
| Korean | c.694G>A | No | - | Baek et al.2012 |
| Korean | c.977G>A | - | - | Kim et al. 2013 |
| Japanese | c.1007delC | - | - | Mutai H et al. 2013 |
| Chinese Han | c.603_604delGG | No | - | Yang et al. 2013 |
| Brazilian | Deletion of the entire POU4F3 gene | No | Vestibular tests shown some degree of visual-vestibular system asymmetry, but the findings are so subtle that are in agreement with the absence of vestibular complains described in other reports | Freitas EL et al. 2014 |
| Chinese Han | c.491C > G | No | - | Wei et al. 2014 |
| Japanese | chr5:145,702,343-145,758,292; 56bp deletion | - | - | Rosenberg 2016 |
| Chinese Han | c.932T>C;c.120+1G>C | No | - | He et al. 2016 |
| Chinese Han | c.337C>T | Yes | Normal vestibular function | Zhang et al. 2016 |
| Chinese Han | c.602delT | - | Normal vestibular function | Cai et al. 2017 |
| Chinese Han | c.982A>G | - | - | Lin et al. 2017 |
| Chinese Han | c.602T>C | No | Normal vestibular function | Gao et al. 2018 |

-: Not mention in the report

**References:**

1. Vahava O, Morell R, Lynch ED, Weiss S, Kagan ME, et al. (1998) Mutation in transcription factor POU4F3 associated with inherited progressive hearing loss in humans. Science 279: 1950-1954.

2. Collin RW, Chellappa R, Pauw RJ, Vriend G, Oostrik J, et al. (2008) Missense mutations in POU4F3 cause autosomal dominant hearing impairment DFNA15 and affect subcellular localization and DNA binding. Hum Mutat 29: 545-554.

3. Pauw RJ, van Drunen FJ, Collin RW, Huygen PL, Kremer H, et al. (2008) Audiometric characteristics of a Dutch family linked to DFNA15 with a novel mutation (p.L289F) in POU4F3. Arch Otolaryngol Head Neck Surg 134: 294-300.

4. de Heer AM, Huygen PL, Collin RW, Kremer H, Cremers CW (2009) Mild and variable audiometric and vestibular features in a third DFNA15 family with a novel mutation in POU4F3. Ann Otol Rhinol Laryngol 118: 313-320.

5. Lee HK, Park HJ, Lee KY, Park R, Kim UK (2010) A novel frameshift mutation of POU4F3 gene associated with autosomal dominant non-syndromic hearing loss. Biochem Biophys Res Commun 396: 626-630.

6. Baek JI, Oh SK, Kim DB, Choi SY, Kim UK, et al. (2012) Targeted massive parallel sequencing: the effective detection of novel causative mutations associated with hearing loss in small families. Orphanet J Rare Dis 7: 60.

7. Kim HJ, Won HH, Park KJ, Hong SH, Ki CS, et al. (2013) SNP linkage analysis and whole exome sequencing identify a novel POU4F3 mutation in autosomal dominant late-onset nonsyndromic hearing loss (DFNA15). PLoS One 8: e79063.

8. Mutai H, Suzuki N, Shimizu A, Torii C, Namba K, et al. (2013) Diverse spectrum of rare deafness genes underlies early-childhood hearing loss in Japanese patients: a cross-sectional, multi-center next-generation sequencing study. Orphanet J Rare Dis 8: 172.

9. Yang T, Wei X, Chai Y, Li L, Wu H (2013) Genetic etiology study of the non-syndromic deafness in Chinese Hans by targeted next-generation sequencing. Orphanet J Rare Dis 8: 85.

10. Freitas EL, Oiticica J, Silva AG, Bittar RS, Rosenberg C, et al. (2014) Deletion of the entire POU4F3 gene in a familial case of autosomal dominant non-syndromic hearing loss. Eur J Med Genet 57: 125-128.

11. Wei Q, Zhu H, Qian X, Chen Z, Yao J, et al. (2014) Targeted genomic capture and massively parallel sequencing to identify novel variants causing Chinese hereditary hearing loss. J Transl Med 12: 311.

12. Rosenberg C, Freitas EL, Uehara DT, Auricchio M, Costa SS, et al. (2016) Genomic copy number alterations in non-syndromic hearing loss. Clin Genet 89: 473-477.

13. He L, Pang X, Chen P, Wu H, Yang T (2016) Mutation in the Hair Cell Specific Gene POU4F3 Is a Common Cause for Autosomal Dominant Nonsyndromic Hearing Loss in Chinese Hans. Neural Plast 2016: 9890827.

14. Zhang C, Wang M, Xiao Y, Zhang F, Zhou Y, et al. (2016) A Novel Nonsense Mutation of POU4F3 Gene Causes Autosomal Dominant Hearing Loss. Neural Plast 2016: 1512831.

15. Cai XZ, Li Y, Xia L, Peng Y, He CF, et al. (2017) Exome sequencing identifies POU4F3 as the causative gene for a large Chinese family with non-syndromic hearing loss. J Hum Genet 62: 317-320.

16. Lin YH, Lin YH, Lu YC, Liu TC, Chen CY, et al. (2017) A novel missense variant in the nuclear localization signal of POU4F3 causes autosomal dominant non-syndromic hearing loss. Sci Rep 7: 7551.

17. Gao X, Xu JC, Wang WQ, Yuan YY, Bai D, et al. (2018) A Missense Mutation in POU4F3 Causes Midfrequency Hearing Loss in a Chinese ADNSHL Family. Biomed Res Int 2018: 5370802.
