# Supplementary material for: Aldh inhibitor restores auditory function in a mouse model of human deafness
Source: PLoS Genet. 2020 Sep 24;16(9):e1009040. doi: 10.1371/journal.pgen.1009040 (PMC7553308; doi:10.1371/journal.pgen.1009040)
Supplement: Attachment — Submitted filename: PG revision Responses to Reviewers.docx [file pgen.1009040.s014.docx]

**Responses to Reviewers’ Comments:**

We’d like to thank the reviewers for positive evaluations and insightful comments on our manuscript.

**Reviewer #1:** Mutations in Pou4f3 are associated with autosomal dominant non-syndromic deafness, DFNA15. The manuscript from Zhu et al. reports finding from a study on two mouse models of DFNA15 used to determine how the pathology occurs and to assess novel therapeutic venues.
Zhu et al. generated a knock-in mouse model mimicking a mutation identified in and Israeli family. This mutation leads to expression of truncated Pou4f3 protein and progressive high to mid frequency hearing loss in heterozygous mice. This loss is associated with stereocilia malformation, mitochondria vacuolization and progressive hair cell loss.
To determine if this pathology is associated with a dominant negative effect of the truncated protein or haplo-insufficiency, the same study is performed in a knock-out model of Pou4f3. Similar outcomes are observed in heterozygous KO mice, suggesting that DFNA15 is associated with haplo-insufficiency.
To explore therapeutic venues, the authors assess one of the pathways that may control Pou4f3 expression: retinoic acid has been shown to suppress Pou4F3. They report modulation of Pou4f3 and recovery of hair cells in vitro (P3 treatment) and hair cells and auditory function up to 9 weeks after 2 to 3 months of age, with daily intraperitoneal injections of DEAB (aldh inhibitor) in knock-in mice.

This work reports important finding that relate to the physiopathology of DFNA15 and provides important results that support the use of retinoic acid inhibitors to regulate pou4f3 expression in heterozygous mutants. The manuscript is clearly written, and the figures well laid out. A few major points highlighted below remain to be addressed.

Major:

- The comparison of the KI het phenotype with the KO het phenotype is meant to confirm that the progression of hearing loss in DFNA15 is associated with haploinsufficiency in the KI het mice. ABR, DPOAEs and ABR peak 1 amplitude are only shown at one time point (3 months of age) for the het KO mice. It would be good to show all three time points or at least the 3- and 7-months data which show, by 7 months of age in the het KI mice, further hearing loss in the mid frequencies.

**Response:** The reviewer raised a valid point. We have now included data (hearing tests and hair cell counts) from 9 months old heterozygous KO mice on a mixed background of C57BL/6J and FVBN (**new S9 and S10B Fig**). The het KO mice displayed hearing loss at multiple frequencies. In addition, we also bred the Pou4f3(Δ/+) mice (KI het) to FVBN background (herein defined mixed background) in order to address the effect of genetic background and environmental insults to hearing loss progression of the DFNA15 mouse model (**new S7 and S8 Fig**). We found that the onset of hearing loss in KI het was significantly delayed on the mixed background (**new S7 Fig**), i.e. between 6-12 months, but was similar to the het KO mice on the same background (9 months, **new S9 Fig**). Furthermore, hearing loss was significantly worse in the young KI het mice after noise exposure, compared to wildtype controls on the mixed background (**new S8 Fig**). These new results highlight the notion that the onset of DFNA15 deafness is modified by genetic and environment factors, consistent with high variability of audiological characteristics among individual DFNA15 patients [1], and may partly explain the lack of auditory phenotype in het Pou4f3 mutant mice reported in previous studies [2, 3].

Thus, in the revised manuscript, we have provided evidence for Pou4f3 haploinsufficiency with het KO mice at two ages (3 and 9 months) and two genetic backgrounds (C57 and mixed). We hope this could address the reviewer’s concern.

- DFNA15 is associated with hearing and balance deficits in patients. Pou4f3 knockout mouse models have been reported to exhibit vestibular dysfunction and display circling behaviors. This aspect, not discussed in the study, is of utmost importance particularly if treatment with adhl inhibitor can also improve balance behavior.

**Response**: We thank reviewer for this insightful comment. We also observed severe vestibular problem with our homozygous KI and KO mice; however, vestibular phenotype was not apparent in the heterozygotes. In fact, in previous clinical reports, vestibular complains were not common among the DFNA15 patients and vestibular tests appeared normal even in patients with such complains (**summarized in** **new S1 Table**). To address this issue carefully, we performed rotarod tests (with 3 test protocols) on 7 months old het KI mice (C57 background) (**new S5 Fig**). We found that while auditory phenotypes in these het KI mice were apparent, they did not exhibit significant balance problem. We also dissect utricles from both wildtype and het KI mice, and found only slight reduction in overall sizes of the utricular sensory epithelia in the het KI mice, without changes in hair cell densities. We have previously shown that age-related vestibular dysfunction occurs much later than hearing loss [4]. We postulate that the het KI mice may develop vestibular deficits at a much older age, and the compensatory effects of central vestibular system and other sensory modalities may explain the mild and inconsistent vestibular impairments observed in both DFNA15 patients and mouse models. Based on this finding, we did not proceed with DEAB treatments to look at the vestibular organ.

- Keithley et al. (Hear Res. 1999 Aug;134(1-2):71-6) have reported that hearing of heterozygote Pou4f3
knockout mice is indistinguishable from that of wild-type animals up to 24 months ruling out haploinsufficiency as a cause for deafness in DFNA15. This result (not mentioned) is in contrast with the report of progressive hearing loss in both mouse models assessed in this current manuscript. Can the author discuss this discrepancy?

**Response**: We agree with the reviewer that this discrepancy is an important issue. We have provided new data to support Pou4f3 haploinsufficiency as the underlying cause (**new S9 Fig**) and that DFNA15 deafness is modified by genetic and environmental factors (**new S7 and S8 Fig**). In view of these new results, we think that such discrepancy could arise from both auditory test methods and genetic background or housing environment of these mutant mice. First, click-evoked ABR recordings are known to be less sensitive in detecting frequency-specific hearing loss [5, 6]. Because the previous two studies only measured click-evoked ABRs [2, 3], it is possible that these heterozygous mice may have also developed high-frequency hearing loss that was not detected by click-evoked ABRs. Second, the mouse strain or sub-strain and the housing environment could also contribute to the onset and progression of auditory phenotypes in these heterozygous Pou4f3 mutants. For example, the 129SvEv strain was known to be exceptionally resistant to hearing loss caused by noise and possibly other insults [7]. Keithley et al. (1999) studied the Pou4f3 mutant mice on a mixed C57BL6/129Sv strain, which may be more resistant to hearing loss. Therefore, a thorough assessment of the previous heterozygous Pou4f3 mutants at various ages with pure-tone ABR tests may help decipher these phenotypic discrepancies.

We have now discussed this issue in detail (lines 260-287).

Minor:

- Supplemental Figure 2: Indicate, on the figure, frequencies assessed for each panels.

**Response**: Done.

- Line 156: Talking about “progression in hearing loss” is confusing here as it is meant to describe hearing improvement.

**Response**: We have rephrased to “deteriorations in auditory functions”, this sentence describes expected progression in hearing loss over time in vehicle-treated mice.

**Reviewer #2:** Zhu et al report on their study characterizing two mouse models of DFNA15. One mouse line has a truncating mutation introduced into the Pou4f3 gene identical to a mutation found in humans that causes autosomal dominant non-syndromic deafness. The other model is knock-out of Pou4f3, and heterozygous mice also have hearing loss, demonstrating the DFNA15 results from haploinsufficiency. The authors go on to show that inhibiting retinoic acid signaling by aldehyde dehydrogenase (Aldh) or RA receptor inhibitors stimulate Pou4f3 expression and prevents hearing loss in the mice. The work shows that DFNA15 results from haploinsufficiency of Pou4f3 and that stimulating Pou4f3 pharmacologically can be therapeutic in mice. This study provides some valuable insights into the Pou4f3 mechanism of hearing loss and generally, the data are convincing. However, there are some issues with the reporting and the comprehensiveness of the datasets that raise some concerns. The authors should take more care in describing the ages that mice were analyzed and the rationale for the selection and also discuss discrepancies and shortcomings. There are also a number of analyses that could be easily performed that would increase the impact of the results. These points are detailed below.

Additional details on the nature of the mice should be included. For the knockin mice, the description and figure S1 leave some ambiguity as to the exact nature of the sequence in the mice. It would be helpful to also include the amino acid sequence of the truncated protein or a cartoon indicating the differences between WT and truncated form.

**Response**: We have now added a figure panel (**new** **S1B Fig**) to illustrate nucleotide and amino acid sequences in wildtype and mutant Pou4f3.

For the knockout mice there is almost no description of how the mouse was generated although it appears that these mice have been previously described in another publication by the authors and this should be cited in the methods (line 228).

**Response**: We have cited our previous paper describing the KO mice in both results (lines 183-185) and methods (line 352).

Data not shown (line 186) should be provided as a supplemental figure. This particular analysis will be of interest considering that it shows lack of consistency with previous published data on known targets of Pou4f3.

**Response**: RT-qPCR analyses of known Pou4f3 genes in wildtype and knockin cochleae have been added (**new S6A Fig**).

The authors should provide a rationale for why RNA-Seq analysis was done on 2 month old mice, which do not yet have detectable hearing deficits. In addition, a file with the raw data used to compile S1 and S2 tables should be included.

**Response**: We collected cochleae samples from both wildtype and Pou4f3(Δ/+) mutant mice at age of 2 months, when the mutant mice started to display hearing loss (**Fig 1C**) but without significant hair cell loss that may confound gene expression analyses. We hypothesize that changes in gene expression should precede functional alterations. We have discussed this rationale in the revised text (lines 140-142).

S1 Table is now re-analyzed using the most recent GO database and revised as **new** **S6B Fig**. Raw data for S6B Fig and other figures were provided in **new** **S1 Data**. The original **S2 table** remains the same, which contains the raw RNA-seq values.

Validation of gene expression changes reported in the knockin mice could be done in the explants tested in Fig. 4 with the DEAB, BMS and RA, would strengthen the mechanistic links.

**Response**: In the revised manuscript, we first validated RNA-seq data and identified *Tmem237* and *Sra1* to be downregulated in the Pou4f3(Δ/+) cochleae (**new S6C Fig**). We then quantified these two genes in DEAB-treated cochlear explants, and showed that *Tmem237* but not *Sra1* was upregulated (**new Fig 4B**). The relevance of these two genes in DFNA15 pathogenesis is current unknown and was discussed in the revised discussion section (lines 298-301). In addition, the effect of DEAB on Pou4f3 expression in explants was strengthened by significant upregulation of Lhx3, a known Pou4f3 target (**new Fig 4C**).

It would be valuable to analyze the level of Pou4f3 and Cyp26a1 in the cochlea of the treated mice shown in Fig. 4G-J) to demonstrate treatment effect on expected target.

**Response**: The reviewer has raised a valid point. We have now quantified the expression of *Cyp26a1*, *Pou4f3* and *Lhx3* by RT-qPCR in Pou4f3(Δ/+) cochleae after DEAB treatment *in vivo* (**new Fig 5A**). DEAB treatment significantly increases the expressions of *Pou4f3* and *Lhx3*, confirming the *in vivo* regulation of Pou4f3. However, *Cyp26a1* expression remained unchanged, possibly due to complex regulatory mechanisms of *Cyp26a1* *in vivo*, a phenotype previously observed with DEAB-treated zebrafish embryos [8]. (lines 230-233)

Why was the 16 kHz region measures in Fig. 4L? The mice, as analyzed in Fig. 2, do not appear to have OHC loss in that region at 4 months (Fig. 2) which is the age that the treated mice would be (4- 5 months if treated at 2-3 months as indicated in methods).

**Response**: Thanks for pointing out this error. We have corrected “16 kHz” to “32 kHz” in the figure legend. Furthermore, we have now collected and added new samples to analysis, and presented histological data for additional frequencies (8, 16 and 32 kHz) in the revised figure (**Fig 5G**).

A discussion of the therapeutic potential of inhibiting RA signaling as a therapeutic strategy and in particular of DEAB and BMS in particular would be informative.

**Response**: This is a valuable suggestion. We have now discussed the potentials and pitfalls of RA signaling and DEAB for future therapeutic applications (lines 311-325).

Numerous issues with grammar and writing style- care should be taken in editing.

Line 25: “…is one of the most common autosomal dominant non-syndromic deafness.” Should be “is one of the most common causes of/forms of autosomal dominant…”

**Response**: Corrected.

Line 217: DNA/RNA sequence should be referred to by 5’ and 3’ end rather than by C-terminus

**Response**: Corrected.

Figure S1: revesion should be reversion

**Response**: Corrected.

Please define all abbreviations used throughout, e.g. FC, padj, dB, SPL, etc.

**Response**: Done.

**Reviewer #3:** Zhu et al have created a knockin mouse model of the first DFNA15 mutation found in humans and demonstrate that the heterozygous mice have a progressive hearing loss similar to the phenotype in affected humans who have a single copy of the dominant mutation. Taken together with the identification similar hearing phenotype in a full knockout Pou4f3 mouse model they suggest this provides confirmation that the mechanism underlying the progressive hearing loss in humans is haploinsufficiency rather than due to a dominant negative action of the mutation. Previous data had suggested that mice heterozygous for Pou4f3 knockout did not have a hearing phenotype. Furthermore, they then use IP delivery of an inhibitor of retinoic acid signalling to ameliorate the progression of the hearing loss in this mouse model suggesting that this may be an approach of therapeutic benefit in humans with DFNA15.
On the whole the dataset is persuasive and raises the exciting possibility of a drug approach to treating an adult onset deafness. However, there are several areas where the manuscript has limitations and should be improved, none of these are major weaknesses that would prevent publication but several do need addressing.

1. Please explain more clearly the nature of the mutation created in the knock-in and the reason for having to introduce a STOP codon. I am guessing this is because the mouse sequence differs to the human one at that base and therefore the 8bp deletion would not create a STOP codon in the mouse sequence but this should be made clear. Does the knock-in fully recapitulate the human mutation with the introduction of the same 4 novel amino acids? This information should be added to FigS1.

**Response**: The reviewer is correct about the rationale of the STOP codon. To help understanding the knockin strategy, we have now added a figure panel (**new** **S1B Fig**) to illustrate nucleotide and amino acid sequences in wildtype and mutant Pou4f3.

2. The manuscript fails to summarise the previous knowledge of DFNA15 or discuss how these findings change our understanding of the effect of this mutation in 2 important respects:
a) There are several previous POU4F3 knock-out mouse models and a spontaneous Dreidel mutant with a 2bp deletion. None of these mice are reported to have a hearing deficit in the heterozygote, although only one has been aged out to 24 months and assessed PMID: 10452377. This is not mentioned in the manuscript. Can the authors suggest a reason for the difference? Are the strain backgrounds the same?

**Response**: The reviewer raised an important issue. We think that the discrepancies in auditory phenotypes of various heterozygous mutants [2, 3] could arise from both auditory test methods and genetic background or housing environments. First, click-evoked ABR recordings are known to be less sensitive in detecting frequency-specific hearing loss [5, 6]. As the previous two studies only measured click-evoked ABRs [2, 3], it is possible that these heterozygous mice may have also developed high-frequency hearing loss that was not detected by click-evoked ABRs. Second, we show that both genetic and environmental factors exhibit significant contributions to the onset and severity of DFNA15 deafness (**new** **S7 and S8 Fig**). The mouse strain or sub-strain and the housing environment could also contribute to the onset and progression of auditory phenotypes in these heterozygous Pou4f3 mutants. For example, the 129SvEv strain was known to be exceptionally resistant to hearing loss caused by noise and possibly other insults [7]. Keithley et al. (1999) studied the Pou4f3 mutant mice on a mixed C57BL6/129Sv strain, which may be more resistant to hearing loss. In another study, Hertzano et al. (2004) reports that “ABR thresholds of age-matched C57BL/6J Pou4f3ddl/+ mice were 35 dB SPL, which is indistinguishable from wild-type mice”. Although the mutant mice were also on C57 background, it is not clear the specific ages when auditory tests were performed. Considering that DFNA15 presents progressive hearing loss and is modifiable by environmental factors, a thorough assessment of the previous heterozygous Pou4f3 mutants at various ages with pure-tone ABR tests may help decipher these phenotypic discrepancies.

We have now discussed this issue in detail (lines 260-287).

b) The second omission is to the work of Weiss et al which suggest that this 8pb del causes a mutant protein which has a longer half-life than the wildtype protein and is localised to the cytoplasm in a cell line model instead of the nucleus. They suggested that this aberrant protein could cause cell death and that this might underlie the hair cell loss in DFNA15 patients. Do the authors consider that this is still a possibility and did they test for where the mutant protein is localised in the knock-in?

**Response**: Previous study suggests that the mutant Pou4f3 neither exhibited transcription activity nor negatively affected the activity of the wildtype Pou4f3 [9]. This is consistent with our data that Pou4f3 haploinsufficiency is the underlying cause of DFNA15 (**Fig 3 and new S9 and S10B Fig**).

To address the reviewer’s question, we examined the subcellular localizations of the wildtype and mutant Pou4f3 in both HeLa cells and hair cells of the knockin mice (**new S1F and S1G Fig**). Unlike the wildtype Pou4f3 protein, which was exclusively localized to the nucleus, the mutant Pou4f3 showed extensive cytoplasmic localization in HeLa cells (**new S1F Fig**), consistent with the previous report [9]. Similarly, we also observed cytoplasmic localization of the mutant Pou4f3 and weaker nuclear Pou4f3 signal in outer hair cells of P10 heterozygous knockin mice (**new S1G Fig**).

The results were described in the text (lines 69-73).

3. Given that previous knock-outs of Pou4f3 have not reported a het phenotype, it is particularly important to be sure that the data from this new model is definitive. Currently, hearing data for only 1 age point is presented for this mouse, at 3 months, when the hearing deficit is modest. Addition of data for 6-7 months would strengthen the case for a het phenotype.

**Response:** Thanks for the suggestion. We have now included data (hearing tests and hair cell counts) from 9 months old heterozygous KO mice on a mixed background of C57BL/6J and FVBN (**new S9 and S10B Fig**). The het KO mice displayed hearing loss at multiple frequencies. In addition, we also bred the Pou4f3(Δ/+) mice (KI het) to FVBN background (herein defined mixed background) in order to address the effect of genetic background and environmental insults to hearing loss progression of the DFNA15 mouse model (**new S7 and S8 Fig**). We found that the onset of hearing loss in KI het was significantly delayed on the mixed background (**new S7 Fig**), i.e. between 6-12 months, but was similar to the het KO mice on the same background (9 months, **new S9 Fig**). Furthermore, hearing loss was significantly worse in the young KI het mice after noise exposure, compared to wildtype controls on the mixed background (**new S8 Fig**). These new results highlight the notion that the onset of DFNA15 deafness is modified by genetic and environment factors, consistent with high variability of audiological characteristics among individual DFNA15 patients [1], and may partly explain the lack of auditory phenotype in het Pou4f3 mutant mice reported in previous studies [2, 3].

Thus, in the revised manuscript, we have provided evidence for Pou4f3 haploinsufficiency with het KO mice at two ages (3 and 9 months) and two genetic backgrounds (C57 and mixed). Again, we sincerely hope this could address the reviewer’s concern.

4. Beginning at line 138, the authors suggest that Pou4f3 is regulated by retinoic acid signalling and has been shown to be supressed by retinoic acid in rat cochlear explants, citing ref 17 for both statements. Ref 17 concerns GATA3 and Prestin expression and does not mention Pou4f3. I have been unable to find another paper describing Pou4f3 being regulated by retinoic acid. Could the authors explain on what they based their rationale for using retinoic acid inhibitors?

**Response**: The paper we referred to (Gross et al. 2011) studied the effects of retinoic acid and other molecules on the expressions of Prestin and selected transcription factors, including Pou4f3 (Brn-3c). Based on Figure 9 of the paper, the author concluded that “at concentrations of 50 and 100 µM, RA significantly decreases prestin and Brn-3c expression in all segments”. Therefore, we hypothesized that downregulating RA signaling may promote the expression of Pou4f3.

5. The authors document several morphological deficits in the DFNA15 model, including OHC loss, stereocilia and mitochondrial deficits but there is an absence of functional assays. Have the authors considered using FM-143 or mitochondrial assays to test functionality on hair cells?

**Response**: Due to the fragility and scarcity of adult hair cells, functional studies of stereocilia and mitochondria remain challenging. We have made attempts to perform FM1-43 dye uptake experiment on adult (7 months old, C57 background) wildtype and heterozygous mutant cochleae (figure below). However, the result is not conclusive due to a couple of issues. First, to ensure viability of hair cells, we can only dissect the apical tip of an adult cochlea. However, this cochlear region corresponds to very low frequencies, where we were unable to record auditory functions. Second, as DFNA15 presents progressive hearing loss, the hair cells at apical tips may still be functional and the FM1-43 data of the apical hair cells may not be informative. Lastly, even the apical region showed rapid degeneration *ex vivo*, bringing difficulties in imaging and analysis. Thus, we decided not to include the FM1-43 data in the revised paper. Nevertheless, genetic mutations or defects are known to cause either stereocilia fusion (Cdc42, SorCS2, Elmod3, Synj2) or elongation (SorCS2, Grxcr2), leading to hearing loss [10-13].

6. Are their balance defects in the 2 mouse models?

**Response**: To address reviewer’s question, we performed rotarod tests (with 3 test protocols) on 7 months old het knock-in mice (C57 background) (**new S5 Fig**). We found that while auditory phenotypes in these het knock-in mice were apparent, they did not exhibit significant balance problem. We also dissect utricles from both wildtype and het knock-in mice, and found slight reduction in overall sizes of the utricular sensory epithelia in the het knock-in mice, without changes in hair cell densities. We have previously shown that age-related vestibular dysfunction occurs much later than hearing loss [4]. We postulate that the het knock-in mice may develop vestibular deficits at a much older age, and the compensatory effects of central vestibular system and other sensory modalities may explain the mild and inconsistent vestibular impairments observed in both DFNA15 patients and mouse models. Indeed, although reported, vestibular complains were not common among the DFNA15 patients and vestibular tests appeared normal even in patients with such complains (**new S1 Table**).

7. Have the RNAseq data been validated by qPCR for any of the genes?

**Response**: From the RNA-seq data, we identified *Tmem237* and *Sra1* to be downregulated in the knock-in cochleae by RT-qPCR (**new S6C Fig**). We also quantified these two genes in DEAB-treated cochlear explants, and showed that *Tmem237* but not *Sra1* was upregulated (**new Fig 4B**). The relevance of these two genes in DFNA15 pathogenesis is current unknown and was discussed in the revised discussion section (lines 298-301).

8. Currently the evidence suggest that DEAB can prevent or slow progression of the hearing loss but it would be interesting to know whether there is an improvement to pre-treatment hearing levels at different ages, suggesting that damaged stereocilia and mitochondrial function can be repaired by Pou4f3 induction. Is this something the authors have assessed?

**Response**: The reviewer raised a question on clinical efficacy of DEAB. Data from the two DEAB treatment paradigms (**Fig 5B-D and Fig 5E**) suggest that DEAB seemed to prevent the progression of hearing loss (or to restore hearing comparing pre- and post-treatment time lines). We hypothesize that early intervention is key to maintain healthy hearing.

9. In the discussion the authors suggest this could form the basis of a therapeutic approach to DFNA15 patients. It would be good to temper this statement with some acknowledgement of the difficulties and barriers to be overcome before this is a viable option.

**Response**: We fully agree with the reviewer. We have now discussed both the potentials and difficulties of RA signaling and DEAB in future therapeutic applications (lines 311-325).

Other points:

Fig S1 reversion is misspelt.

**Response**: Corrected.

Define abbreviations, FDA, H&E …

**Response**: Done.

Fig 2 % OHC loss- please define what the percentage is of?

**Response**: % OHC loss was defined as number of OHC missing divided by total OHCs demarcated by F-actin stainings. The missing OHC can be easily distinguished by lack of Myo7a signal and presence of remaining F-actin rings at reticular lamina. We added the definition in the revised methods.

Fig2 and Fig3 for clarity please put the ages on the charts rather than the legends.

**Response**: Done.

Fig 4A-D. What is the assay? qPCR? What is FC?

**Response**: mRNA expression was quantified by RT-qPCR. We’ve also spelt out FC as “fold change” in the figure.

Fig 4E&F. Please quantify the mutant and wildtype protein separately, there seems to be a difference in effect.

**Response**: Thank you for the critical comment. We have now quantified wildtype and mutant Pou4f3 protein separately (Fig 4H and 4I). Indeed, DEAB promoted expression of wildtype Pou4f3 protein, but not mutant Pou4f3 protein, in cochlear explants from the *Pou4f3(Δ/+)* mice. It has been reported that the mutant Pou4f3 display long half-life than the wildtype Pou4f3 [14], which may confound the effects of DEAB. We have revised the text accordingly (lines 222-225).

Fig 4G-I. Please clarify whether the ABRs were immediately after 3 weeks’ treatment or 3 weeks after the treatment finished.

**Response**: ABRs were performed after start of the 3-week treatment. We have clarified this in the text (lines 234-235): “We then examined the *in vivo* physiological effects of DEAB treatment with DPOAE and ABR tests performed 1 day after last vehicle or DEAB injections.”

The authors should consider depositing their RNAseq data in the G-EAR portal.

**Response**: We thank reviewer for the suggestion. We are now performing additional RNA-seq experiments on other ages, and will consider depositing to G-EAR portal after we collected these new data. The RNA-seq reported in this manuscript has been deposited to GEO database with accession number GSE139145 (described in methods).

**References for reviewers:**

1.	Frydman M, Vreugde S, Nageris BI, Weiss S, Vahava O, Avraham KB. Clinical characterization of genetic hearing loss caused by a mutation in the POU4F3 transcription factor. Archives of otolaryngology--head & neck surgery. 2000;126(5):633-7. PubMed PMID: 10807331.

2.	Keithley EM, Erkman L, Bennett T, Lou L, Ryan AF. Effects of a hair cell transcription factor, Brn-3.1, gene deletion on homozygous and heterozygous mouse cochleas in adulthood and aging. Hear Res. 1999;134(1-2):71-6. Epub 1999/08/19. doi: S0378-5955(99)00070-2 [pii]. PubMed PMID: 10452377.

3.	Hertzano R, Montcouquiol M, Rashi-Elkeles S, Elkon R, Yucel R, Frankel WN, et al. Transcription profiling of inner ears from Pou4f3(ddl/ddl) identifies Gfi1 as a target of the Pou4f3 deafness gene. Hum Mol Genet. 2004;13(18):2143-53. Epub 2004/07/16. doi: 10.1093/hmg/ddh218

ddh218 [pii]. PubMed PMID: 15254021.

4.	Wan G, Ji L, Schrepfer T, Gong S, Wang GP, Corfas G. Synaptopathy as a Mechanism for Age-Related Vestibular Dysfunction in Mice. Front Aging Neurosci. 2019;11:156. Epub 2019/07/12. doi: 10.3389/fnagi.2019.00156. PubMed PMID: 31293415; PubMed Central PMCID: PMCPMC6606700.

5.	Fausti SA, Frey RH, Henry JA, Olson DJ, Schaffer HI. Early detection of ototoxicity using high-frequency, tone-burst-evoked auditory brainstem responses. Journal of the American Academy of Audiology. 1992;3(6):397-404. PubMed PMID: 1486202.

6.	Stapells DR. Low-Frequency Hearing and the Auditory Brainstem Response. American journal of audiology. 1994;3(2):11-3. doi: 10.1044/1059-0889.0302.11. PubMed PMID: 26661602.

7.	Yoshida N, Hequembourg SJ, Atencio CA, Rosowski JJ, Liberman MC. Acoustic injury in mice: 129/SvEv is exceptionally resistant to noise-induced hearing loss. Hear Res. 2000;141(1-2):97-106. Epub 2000/03/14. doi: 10.1016/s0378-5955(99)00210-5. PubMed PMID: 10713498.

8.	White RJ, Nie Q, Lander AD, Schilling TF. Complex regulation of cyp26a1 creates a robust retinoic acid gradient in the zebrafish embryo. PLoS Biol. 2007;5(11):e304. Epub 2007/11/23. doi: 10.1371/journal.pbio.0050304. PubMed PMID: 18031199; PubMed Central PMCID: PMCPMC2080651.

9.	Walters BJ, Coak E, Dearman J, Bailey G, Yamashita T, Kuo B, et al. In Vivo Interplay between p27(Kip1), GATA3, ATOH1, and POU4F3 Converts Non-sensory Cells to Hair Cells in Adult Mice. Cell Rep. 2017;19(2):307-20. Epub 2017/04/14. doi: 10.1016/j.celrep.2017.03.044. PubMed PMID: 28402854; PubMed Central PMCID: PMCPMC5423718.

10.	Ueyama T, Sakaguchi H, Nakamura T, Goto A, Morioka S, Shimizu A, et al. Maintenance of stereocilia and apical junctional complexes by Cdc42 in cochlear hair cells. Journal of cell science. 2014;127(Pt 9):2040-52. Epub 2014/03/13. doi: 10.1242/jcs.143602. PubMed PMID: 24610943.

11.	Forge A, Taylor RR, Dawson SJ, Lovett M, Jagger DJ. Disruption of SorCS2 reveals differences in the regulation of stereociliary bundle formation between hair cell types in the inner ear. PLoS Genet. 2017;13(3):e1006692. Epub 2017/03/28. doi: 10.1371/journal.pgen.1006692. PubMed PMID: 28346477; PubMed Central PMCID: PMCPMC5386298.

12.	Li W, Feng Y, Chen A, Li T, Huang S, Liu J, et al. Elmod3 knockout leads to progressive hearing loss and abnormalities in cochlear hair cell stereocilia. Hum Mol Genet. 2019;28(24):4103-12. Epub 2019/10/20. doi: 10.1093/hmg/ddz240. PubMed PMID: 31628468; PubMed Central PMCID: PMCPMC7305813.

13.	Manji SS, Williams LH, Miller KA, Ooms LM, Bahlo M, Mitchell CA, et al. A mutation in synaptojanin 2 causes progressive hearing loss in the ENU-mutagenised mouse strain Mozart. PLoS One. 2011;6(3):e17607. Epub 2011/03/23. doi: 10.1371/journal.pone.0017607. PubMed PMID: 21423608; PubMed Central PMCID: PMCPMC3057978.

14.	Weiss S, Gottfried I, Mayrose I, Khare SL, Xiang M, Dawson SJ, et al. The DFNA15 deafness mutation affects POU4F3 protein stability, localization, and transcriptional activity. Molecular and cellular biology. 2003;23(22):7957-64. PubMed PMID: 14585957; PubMed Central PMCID: PMC262385.
