# Supplementary material for: Aldh inhibitor restores auditory function in a mouse model of human deafness
Source: PLoS Genet. 2020 Sep 24;16(9):e1009040. doi: 10.1371/journal.pgen.1009040 (PMC7553308; doi:10.1371/journal.pgen.1009040)
Supplement: Attachment — Submitted filename: Reviewer 3.docx [file pgen.1009040.s015.docx]

Reviewer 3 – revised version.

Dear Editor,

The authors have addressed most of my concerns in my first review by the addition of a lot of new data and also widening the discussion to make the relevance of these data to the field much clearer. In particular, the inclusion of auditory phenotype from mice of a mixed background is a very good addition. I have 2 requests for the revised manuscript.

Comment R1. Could the authors confirm that whether the hom mutants have a hearing loss on the mixed strain background preferably by including ABR data for these mice. As the het mutants have a different phenotype on this background it is important to know the phenotype of the homs.

Comment R2. The authors replied to my query 4 in the original review:

*Beginning at line 138, the authors suggest that Pou4f3 is regulated by retinoic acid*

*signalling and has been shown to be supressed by retinoic acid in rat cochlear explants,*

*citing ref 17 for both statements. Ref 17 concerns GATA3 and Prestin expression and*

*does not mention Pou4f3. I have been unable to find another paper describing Pou4f3*

*being regulated by retinoic acid. Could the authors explain on what they based their*

*rationale for using retinoic acid inhibitors?*

***Response****: The paper we referred to (Gross et al. 2011) studied the effects of retinoic*

*acid and other molecules on the expressions of Prestin and selected transcription*

*factors, including Pou4f3 (Brn-3c). Based on Figure 9 of the paper, the author concluded*

*that “at concentrations of 50 and 100 μM, RA significantly decreases prestin and Brn-3c*

*expression in all segments”. Therefore, we hypothesized that downregulating RA*

*signaling may promote the expression of Pou4f3.*

I am perplexed by this response as the paper by Goss et al. 2011 (cited below as ref 17 in the original submission and 24 in this revision) only has 6 figures, there is no figure 9. It contains no mention of Pou4f3 or Brn-3c throughout. Have the authors cited the wrong paper? Please clarify as this is the only basis for their rationale that retinoic acid regulates Pou4f3 and the use of manipulators.

*24. Gross J, Stute K, Fuchs J, Angerstein M, Amarjargal N, Mazurek B. Effects of retinoic acid and butyric acid on the expression of prestin and Gata-3 in organotypic cultures of the organ of corti of newborn rats. Dev Neurobiol. 2011;71(7):650-61. Epub 2011/02/24. doi: 10.1002/dneu.20881. PubMed PMID: 21344672.*
