# Supplementary material for: Aldh inhibitor restores auditory function in a mouse model of human deafness
Source: PLoS Genet. 2020 Sep 24;16(9):e1009040. doi: 10.1371/journal.pgen.1009040 (PMC7553308; doi:10.1371/journal.pgen.1009040)
Supplement: Attachment — Submitted filename: PG revision2 Responses to Reviewers.docx [file pgen.1009040.s016.docx]

**[Revision 2]**

**Responses to Reviewers’ Comments:**

We’d like to again thank the reviewers for positive evaluations and insightful comments on our manuscript.

**Reviewer #1:** The authors have substantially revised the manuscript and included new data that address this reviewer's concerns. In particular they have now included new data that highlight effect of genetic background and environmental insults to hearing loss progression associated with DFNA15. They have also now included rotarod tests that demonstrate that vestibular defects are not observed in heterozygous KI mice by 7 months of age.

The manuscript is greatly improved from its original version and is worthy of publication in PLOS Genetics

**Response**: Thank you!

**Reviewer #2:** This revised manuscript has addressed most of my previous concerns and is much improved. My only remaining concern is the interpretation of some of the results relating to the Pou4f3 -/+ mice as compared to the Δ/+ mice and the strong conclusions that the similarity between the mice is conclusive evidence that dominant mutations in Pou4f3 cause hearing loss as a result of haploinsufficiency.

The statement on line 187 is misleading, “The heterozygous knockout Pou4f3(-/+) mice displayed similar auditory phenotypes as the Pou4f3(Δ/+) mice (Fig 3)”. The auditory phenotype of the Pou4f3 (-/+) mice is not similar to the Pou4f3(Δ/+) mice. It appears to be less severe. This point is important as the manuscript is making a claim that Pou4f3 mutations cause hearing loss as a result of haploinsufficiency, which is in conflict with other mouse models and findings in the field. Thus, it is critical that the authors report on the results very precisely without over-stating the conclusions and interpretations.

One thing to consider is that possibility that both haploinsufficiency and a dominant negative activity are at play. The relatively protracted phenotype of the -/+ mice may indicate that haploinsufficiency is the cause of age-related high-frequency hearing loss in the mice with a complete loss of one copy of the gene whereas a dominant negative phenotype is the cause of the more severe hearing loss in the Δ/+ mice, where generation of a truncated protein may interfere with the function of the wild-type protein, for example.

**Response**: We thank the reviewer for favorable comments and the concern raised. We believe that Pou4f3 haploinsufficiency is the main cause of DFNA15 deafness as discussed in the revision 1 of the manuscript, and the lack of reported phenotypes in previous models could be addressed with thorough auditory tests at different ages, on different genetic backgrounds and using pure-tone tests. However, we do agree with the reviewer that the potential effect of mutant Pou4f3 protein cannot be completely excluded. As such, we have carefully revised the paper to reflect such possibility.

Specific changes are appended below: [added text]

Abstract: …DFNA15 is [likely] caused by haploinsufficiency… *Pou4f3* haploinsufficiency as the [main] underlying cause…

Lines 54, 203 and 259: … *Pou4f3* haploinsufficiency as the [main] underlying cause

The misleading statement on line 187 (revision 1) has been removed as the results following are sufficiently explanatory.

Revised text (Lines 273-279): In this study, we found that both *Pou4f3(Δ/+)* knockin and *Pou4f3(-/+)* knockout mouse models displayed hearing loss on both C57BL/6J and mixed genetic backgrounds, suggesting that *POU4F3* haploinsufficiency is sufficient to cause hearing loss reported in the DFNA15 patients. As the auditory phenotypes of *Pou4f3(-/+)* knockout appeared to be milder than the *Pou4f3(Δ/+)* knockin mice, it remains a possibility that the mutant Pou4f3 may serve a negative role to exacerbate the effects of *Pou4f3* haploinsufficiency on hair cell survival and hearing loss.

**Reviewer #3:** The authors have addressed most of my concerns in my first review by the addition of a lot of new data and also widening the discussion to make the relevance of these data to the field much clearer. In particular, the inclusion of auditory phenotype from mice of a mixed background is a very good addition. I have 2 requests for the revised manuscript.

Comment R1. Could the authors confirm that whether the hom mutants have a hearing loss on the mixed strain background preferably by including ABR data for these mice. As the het mutants have a different phenotype on this background it is important to know the phenotype of the homs.

**Response**: Indeed, we have previously looked into this issue and collected the data, which is now include in the revision as **new S7A and S7B Fig**. Briefly, we performed DPOAE and ABR tests on wildtype (n=18), heterozygous (n=21) and homozygous (n=4) mutants bred on the mixed background, and found that the homozygotes were completely deaf by 3 weeks age. The results indicate that the essential roles of Pou4f3 in hair cell survival and function were independent of genetic backgrounds.

Comment R2. The authors replied to my query 4 in the original review:

*Beginning at line 138, the authors suggest that Pou4f3 is regulated by retinoic acid signalling and has been shown to be supressed by retinoic acid in rat cochlear explants, citing ref 17 for both statements. Ref 17 concerns GATA3 and Prestin expression and does not mention Pou4f3. I have been unable to find another paper describing Pou4f3 being regulated by retinoic acid. Could the authors explain on what they based their rationale for using retinoic acid inhibitors?*

*Response: The paper we referred to (Gross et al. 2011) studied the effects of retinoic acid and other molecules on the expressions of Prestin and selected transcription factors, including Pou4f3 (Brn-3c). Based on Figure 9 of the paper, the author concluded that “at concentrations of 50 and 100 μM, RA significantly decreases prestin and Brn-3c expression in all segments”. Therefore, we hypothesized that downregulating RA signaling may promote the expression of Pou4f3.*

I am perplexed by this response as the paper by Goss et al. 2011 (cited below as ref 17 in the original submission and 24 in this revision) only has 6 figures, there is no figure 9. It contains no mention of Pou4f3 or Brn-3c throughout. Have the authors cited the wrong paper? Please clarify as this is the only basis for their rationale that retinoic acid regulates Pou4f3 and the use of manipulators.

*24. Gross J, Stute K, Fuchs J, Angerstein M, Amarjargal N, Mazurek B. Effects of retinoic acid and butyric acid on the expression of prestin and Gata-3 in organotypic cultures of the organ of corti of newborn rats. Dev Neurobiol. 2011;71(7):650-61. Epub 2011/02/24. doi: 10.1002/dneu.20881. PubMed PMID: 21344672.*

**Response**: We sincerely apologize for the mistake in citing the wrong paper. We have corrected the reference in the revised manuscript. The corrected reference should be:

*24.Gross J, Angerstein M, Fuchs J, Stute K, Mazurek B. Expression analysis of prestin and selected transcription factors in newborn rats. Cell Mol Neurobiol. 2011;31(7):1089-101. Epub 2011/05/27. doi: 10.1007/s10571-011-9708-z. PubMed PMID: 21614551.*
